# User experience and satisfaction with specialty consultations and surgical care in secondary and tertiary level hospitals in Mexico

**DOI:** 10.1186/s12913-019-4706-9

**Published:** 2019-11-21

**Authors:** Svetlana V. Doubova, Claudia Infante-Castañeda, Sanam Roder-DeWan, Ricardo Pérez-Cuevas

**Affiliations:** 1 Epidemiology and Health Services Research Unit CMN Siglo XXI, Mexican Institute of Social Security, Av. Cuauhtemoc 330, Zip Code: 06720 Mexico City, Mexico; 2 Institute of Social Research, National Autonomous University of Mexico, Mexico City, Mexico; 3 Ifakara Health Institute, Dar es Salaam, Tanzania; 4 Department of Global Health and Population, Harvard T.H. Chan School of Public Health, 665 Huntington Avenue, Boston, MA 02115 USA; 5 Center for Health Systems Research, National Institute of Public Health, Universidad No. 655 Colonia Santa María Ahuacatitlán, Zip Code: 62100 CuernavacaCity, Mexico; 6 Division of Social Protection and Health, Jamaica Country Office, InterAmerican Development Bank, 6 Montrose road, Kingston, Jamaica

**Keywords:** User experience, User satisfaction, Outpatient consultations, Surgical care, México

## Abstract

**Background:**

To evaluate the association between user experience and satisfaction with specialty consultations and surgical care at the Mexican Institute of Social Security (IMSS) secondary and tertiary level hospitals.

**Methods:**

We conducted secondary data analysis of the cross-sectional 2017 IMSS National Satisfaction Survey. The dependent variables were user satisfaction with outpatient consultation and with surgery. The study’s independent variables were user experience with these services. The Lancet Global Health Commission on High Quality Health Systems in the Sustainable Development Era framework was used to guide the analysis. For each dependent variable a double-weighted Poisson regression model with robust variance was performed and considered clustering of the observations within 111 secondary level and 25 tertiary level hospitals.

**Results:**

The study included 6713 outpatient consultation users and 528 surgery users. 83% of users attending outpatient consultations and 86.6% of users who underwent inpatient surgery at IMSS hospitals were satisfied with the service received. The common patient negative experiences with specialty consultations and surgical care were long waiting time (40%) and lack of hospital cleanliness (20%). An additional concern was the lack of clinical examination during the consultation (25%). Shorter waiting times, health provider courtesy, good communication, clinical examination, and hospital cleanliness were associated with patient satisfaction with specialty consultations. Having the surgery without prior postponement(s) and without complications increased the probability of patient satisfaction.

**Conclusion:**

Patient satisfaction with hospital outpatient consultations and surgical care may be raised by focusing on improvement strategies to enhance positive patient experiences with care.

## Background

In the last decades, health service user experience and satisfaction surveys have been extensively used worldwide. These surveys provide valuable information on health service quality to inform decision-makers, healthcare providers and the public to guide quality improvement initiatives [1]. Repeated surveys serve to evaluate the effectiveness of new healthcare policies and programs and improve accountability through open access to the survey results, internal feedback and benchmarking with similar healthcare providers [1].

The value of user satisfaction surveys has been proven by previous research that shows a link between better clinical (e.g., technical aspects of the process of care) and non-clinical quality of care (e.g., interpersonal care, quality of basic amenities, etc.) and higher patient satisfaction [2–6].

Patient satisfaction with healthcare is a complex construct that reflects a subjective evaluation of health services and providers based on personal preferences and expectations and actual experiences with care [7–9]. These experiences include technical and interpersonal aspects of healthcare, accessibility, affordability, acceptability, quality of the infrastructure and equipment at health facilities, and health outcomes. Also, patients’ experiences and satisfaction with healthcare should consider person-related characteristics as potential determinants and confounders simultaneously [6], given that multiple studies found that male sex, older age, lower levels of schooling and better self-perceived health status were associated with higher patient satisfaction [9–13].

International research has reported important differences in satisfaction and experiences with healthcare in low and high-income countries. The 2005–2012 Gallup World Polls revealed that in Sub-Saharan Africa only 42% of respondents were satisfied with the availability of high-quality care, compared to 86% in Northern Europe [14]. There are multiple common and specific patient experiences among countries and healthcare providers at different levels (e.g., individual, community, institutional, primary or secondary levels) that determine satisfaction. The general population survey of the World Health Organization in 41 countries identified prompt attention, improved health outcomes, dignity and effective patient-provider communication as the most important characteristics of healthcare [15].

Hospital care comprises specialized outpatient and in-patient healthcare for people whose medical conditions cannot be resolved at the primary care level. Patient satisfaction with hospital care varies among providers within and among countries. For instance, in the United States [16], the Hospital Consumer Assessment of Healthcare Providers and Systems survey found high levels of satisfaction with care; 63% of patients rated their care as high (9 or 10 points out of 10), 26% as 7 or 8, whereas only 11% gave a rating of 6 or less. However, this survey found that private or public not-for-profit hospitals were rated higher than for-profit hospitals (64.8 and 65.4% vs. 59.1% respectively). Also, hospitals within a top quartile of nurses-to-patient-days ratio were rated higher than those in the bottom quartile (70.2% vs.63.5%). At the same time, in Peru, patient satisfaction ranged from 25 to 62.1% among different hospitals [17–19].

During the last five years in Mexico, patient satisfaction surveys have been routinely used. Yet, the relationship between patient experiences and satisfaction with hospital care is poorly understood. Our literature review revealed only data from the Mexican Survey of Health and Nutrition [20, 21] and a 2004 study from 15 hospitals in the state of Hidalgo [22]. Both sources reported 80% or higher satisfaction of patients with hospital care. These studies identified that the primary experiences related to dissatisfaction were lack of disease-related information, unfriendly physician attitude, lack of restrooms in the waiting area and appearance of clinical complications.

The Mexican Institute of Social Security (IMSS) is the largest healthcare provider in Mexico. In 2016, IMSS had 1506 primary care clinics, 247 secondary level, and 36 tertiary level hospitals and it provided care for 63,480,327 affiliates (50% of the Mexican population) [23]. On an average day, IMSS delivers 78,000 specialty consultations and 4000 surgeries. Since 2009, the IMSS National Satisfaction Survey has been carried out on a regular basis; however, its data have not been analyzed in-depth to identify the determinants of patient satisfaction and guide the design of quality improvement strategies. Therefore, the objective of this study was to evaluate the association between user experience and satisfaction with specialty consultations and surgical care at IMSS secondary and tertiary level hospitals. We hypothesized that specific positive user experiences are associated with their satisfaction with care.

## Methods

This study is a secondary data analysis of the cross-sectional November 2017 IMSS National Satisfaction Survey (ENSAT).

The 2017 ENSAT was conducted with users attending to medical care at facilities of any of the three levels of care (primary, secondary and tertiary), and its design used a two-stage stratified probabilistic sampling for each level of care. Our study analyzed the information for the outpatient specialty consultations and inpatient surgeries at IMSS secondary and tertiary care hospitals. In the first stage, the ENSAT sampling frame included a complete list of IMSS hospitals. The hospitals were stratified by district (IMSS is divided into 35 districts in 32 States) and level of care. Subsequently, within each district, the hospitals were selected based on probability proportional to their average number of daily specialty consultations and inpatient surgeries. In the second stage, the sampling frame for patients in each hospital was based on the list of patients who had specialty consultation or were discharged from the surgery ward. Patient selection was through systematic sampling with a random starting point and a fixed periodic interval. In each hospital, this interval was contingent on the average number of consultations or surgeries per day.

The sample size considered the absolute allowable error in the estimation of 1.1% in secondary care hospitals and 1.5% in tertiary care hospitals. The design effect ranged between 1.1 and 2.7 in secondary care hospitals and between 1.1 and 1.4 in tertiary care hospitals. The expansion factors were calculated as the inverse of the selection probability of each sampling unit to represent the population of IMSS daily users.

A private firm (Berumen and Associates) conducted the survey. Trained interviewers carried out direct, structured interviews with IMSS health services users ≥18 years of age. The interviewers used a satisfaction questionnaire that the Center for Evaluation Research and Surveys of the National Institute of Public Health had previously validated: this included content validation by the expert group. According to this validation, the questionnaire has good content validity; yet, the results were not published in academic or other publicly available sources.

The response rate in the survey was 85% for secondary care hospitals, and 83% for tertiary care hospitals. At the secondary level of care, the final sample of the survey included 111 hospitals and 4625 users of outpatient consultation with specialists and 380 users who underwent inpatient surgery. At the tertiary level of care, the sample included 25 hospitals and 2458 users of specialty consultations and 196 users of inpatient surgery.

### Study variables

The dependent variable was user satisfaction with healthcare. This variable was measured through a single question with seven response options: very satisfied, satisfied, neither satisfied nor dissatisfied, dissatisfied, very dissatisfied, not sure, decline to answer. These options were then grouped into two categories: satisfied (very satisfied, satisfied) and not satisfied (dissatisfied, very dissatisfied and neither satisfied nor dissatisfied). The last category was included in the “not satisfied” group as from our perspective those who answered that they were “neither satisfied nor dissatisfied” (4.7% of outpatient consultation and 3.8% of surgical services users) were unable to classify as satisfactory their experience with the health services. In addition, we did not include in the analysis those users who were “not sure” or declined to answer (0.1%: 7 users of outpatient consultation and one surgery user).

The study’s independent variables were user experiences with outpatient consultation and user experience with surgical care, measured on a dichotomous scale (yes and no). We organized the existing survey variables on user experiences into five groups according to the 2018 Lancet Global Health Commission on High-Quality Health Systems conceptual framework [24], as follows (Table 1).

**Table 1:** User experience with specialty consultations and surgical care organized under the Lancet Global Health Commission on High-Quality Health Systems conceptual framework

Domains	Specialty consultation	Surgery
I. Client focus	Waiting time for outpatient consultation in hospital outpatient area ≤ 30 min	Waiting time between referral to surgery and actual surgery is ≤20 completed waiting days History of surgery postponement(s)
II. Respect	Courtesy: Specialist greeted the patient at the beginning of the consultation Clear communication: Specialist gave the patient an opportunity to talk about health-related concerns Specialist listened to the patient with attention and without interruptions Specialist clearly answered patient questions Specialist resolved patient doubts about health-related self-care	Courtesy: Surgeon greeted patient before surgery Clear communication: Surgeon explained the risks and benefits of surgery Surgeon gave clear information to the patient’s relatives
III. Competent care	Specialist performed clinical examination	
IV. Quality of basic amenities	Hospital cleanliness was very good or good	Hospital cleanliness was very good or good
V. Quality impact	Quality impact on financial risk protection: Patient received all prescribed medicines in the hospital pharmacy free due to their coverage by IMSS health insurance (reference group did not have any prescription to fulfill)	Quality impact on health: Patient did not have complications that required another surgery

The analysis included the following patient socio-demographic and clinical covariates: sex (female, male); age groups (≤35 years; > 35 & ≤ 44 years; > 44 & ≤ 64 years; ≥65 years); schooling (incomplete elementary school or without formal education; complete elementary school or incomplete secondary school; complete secondary school or higher); region of residence (divided in seven strata from stratum 1 (lowest socioeconomic level) to 7 (highest socioeconomic level) [25]; the level of care in which service was received (secondary, or tertiary).

For patients that attended the outpatient consultation, we also analyzed the type of specialty consultation: medical-surgical, or clinical specialties, and causes of specialty consultation that were grouped according to the International Classification of Diseases 10th revision (ICD-10) code and its frequencies, with low frequency codes grouped in the “others diseases” category: (1) Neoplasms; 2) Endocrine, nutritional and metabolic diseases; 3) Mental, behavioural disorders and diseases of the nervous system; 4) Diseases of the eye and adnexa; 5) Diseases of the respiratory system and diseases of the ear; 6) Diseases of the circulatory system; 7) Diseases of the digestive system; 8) Diseases of the musculoskeletal system and connective tissue; 9) Diseases of the genitourinary system; 10) Pregnancy, childbirth and the puerperium; 11) Injury, poisoning and other consequences of external causes; 12) Others diseases; 13) Factors influencing health status and contact with health services; 14) Symptoms, signs and abnormal clinical and laboratory findings, not elsewhere classified).

For patients with surgery, we included causes of surgery variable: 1) Diseases of the digestive system; 2) Diseases of the genitourinary system; 3) Diseases of the musculoskeletal system and connective tissue; 4) Diseases of the eye and adnexa; 5) Injury, poisoning and other consequences of external causes; 6) Other types of diseases and cesarean section; 7) Symptoms, signs and abnormal clinical and laboratory findings, not elsewhere classified.

We included in our analysis causes of specialty consultation and of surgery as approximation to the patient health status because previous research has shown that patient health status is an important potential determinant of satisfaction [6].

### Statistical analysis

We used descriptive statistics to analyze patient characteristics and experiences with healthcare. We performed a bivariable analysis, including chi-square tests between the dependent variable (user satisfaction) and each independent variable (user experiences), or categorical covariates.

For each model (satisfaction with outpatient consultation or with surgical care) we performed a double-weighted Poisson regression model with robust variance. We used Poisson regression with robust variance as recommended for cross-sectional studies with high-prevalence binary outcomes [26]. We applied a double-weighted strategy with the use of survey weights and stabilized inverse probability (IP) weights to adjust the analysis for sample weights and to correct for potential missing data bias [27]. The number of participants with one or more missing values in the study variables was 370 (5.2%, *N* = 7083) for specialty consultation and 48 (8.3%, *N* = 576) for surgery. The IP-weights technique consists of assigning a weight to each person with complete information so that, in the analysis, they are accounting for themselves as well as for those with similar characteristics who had missing information. It allows for conditional exchangeability (within the level of measured covariates) of those without and with missing data. The denominator for IP-weights was the probability of having missing data conditional on the following covariates: sex, age, stratum of the region of residence, level of care and ICD code for the cause of specialty consultation (or cause of surgery). The numerator was the probability of “having missing data” regardless of the covariates. The multiple Poisson regression model included the dependent variable, all conceptually relevant independent variables, and covariates found in prior research related to user satisfaction and available in the ENSAT databases. Previous to the multiple regression model, we calculated crude prevalence ratio (PR) and 95% confidence interval (CI) for each independent variable and each covariate.

Furthermore, given that ENSAT 2017 included 111 secondary level and 25 tertiary level hospitals, we put together data of the secondary and tertiary level hospitals; we included in the analysis the variable that specifies level of care and we used the “vce (cluster hospital)” command for the robust variance to explicitly state that observations were grouped within the hospitals. This decision was supported by the fact that differences in infrastructure and staff at IMSS are primarily defined by the level of care (general hospitals provide a secondary level of care and highly specialized hospitals provide a tertiary level of care).

In addition, we performed sensitivity analyses. We conducted multilevel double-weighted Poisson regressions, using random effects for hospitals, to account for the complex structure of the data with patients nested within facilities with ENSAT 2017 and 2016 data. All analyses were performed using STATA 14 software and considering estimates with *p* < 0.05 to be statistically significant.

## Results

Table 2 describes the socio-demographic, clinical characteristics and healthcare experiences of users attending outpatient consultations with specialists at IMSS hospitals**.** Out of the 6713 users, most were women (69.6%); 23.7% of the respondents were younger than 36 years of age, 54.7% were between 36 and 64 years and 21.6% were older; with completed secondary school or higher education (65.1%); and residents of the region of stratum four and six that correspond to medium and high socio-economic levels. The majority (83.3%) attended their consultation with a specialist at a secondary level hospital; 56.4% had a medical-surgical consultation, mostly due to diseases of the genitourinary system (11.6%), injury, poisoning and other consequences of external causes (11%), followed by neoplasms (8.3%) and diseases of the musculoskeletal system and connective tissue (8.2%).

**Table 2:** Socio-demographic, clinical characteristics and healthcare experiences of users attending outpatient consultations with specialists

Variables	Total *n* = 6713 Weighted %
Socio-demographic and clinical characteristics	
Women	69.6
Age groups	
≤ 35 years	23.7
> 35 & ≤44 years	16.7
> 44 & ≤64 years	38.0
≥ 65 years	21.6
Schooling	
Incomplete elementary school or without formal education	12.5
Compete elementary school	22.4
Complete secondary school or higher	65.1
Region of residence according to the socio-economic level	
Stratum 1 (lowest socio-economic level)	3.8
Stratum 2	12.2
Stratum 3	10.6
Stratum 4	21.1
Stratum 5	13.3
Stratum 6	21.4
Stratum 7 (highest socio-economic level)	17.6
Level of healthcare in which service was received	
Secondary	83.3
Tertiary	16.7
Type of specialty consultation	
Medical-surgical consultation	56.4
Clinical specialties consultation	43.6
Cause of specialty consultation	
Diseases of the genitourinary system	11.6
Injury, poisoning and other consequences of external causes	11.0
Neoplasms	8.3
Diseases of the musculoskeletal system and connective tissue	8.2
Diseases of the circulatory system	7.7
Endocrine, nutritional and metabolic diseases	7.1
Diseases of the eye and adnexa	6.4
Diseases of the respiratory system and diseases of the ear	5.3
Pregnancy, childbirth and the puerperium	5.5
Mental, behavioural disorders and diseases of the nervous system	4.6
Diseases of the digestive system	3.8
Other diseases	5.4
Factors influencing health status and contact with health services	7.2
Symptoms, signs and abnormal clinical and laboratory findings, not elsewhere classified	7.9
Users experience with outpatient consultation	
I. Client focus	
Waiting time ≤ 30 min	59.2
II. Respect	
Specialist greeted patient at the beginning of the consultation	81.6
Specialist gave the patient an opportunity to talk about health-related concerns	87.9
Specialist listened to the patient with attention and without interruptions	89.4
Specialist answered clearly patient’s questions	90.7
Specialist resolved patient’s doubts about health-related self-care	80.9
III. Competent care	
Specialist performed clinical examination	75.0
IV. Quality of basic amenities	
Hospital’s cleanliness	
Good, very good	78.3
Regular, bad or terrible	21.7
V. Quality impact on financial risk protection	
Patients received all prescribed medicines in the hospital pharmacy	
Yes	47.2
No	2.8
Did not have any prescription to fill	50.0
Overall satisfaction	
Satisfied with consultation	83.0

Regarding the experience of patients attending outpatient consultations with a specialist: 59.2% of respondents waited for a consultation for 30 min or less; 81.6% reported that the specialist greeted them at the beginning of the consultation; 87.9% had the opportunity to talk about their health-related concerns; 89.4% mentioned that the specialist listened to them with attention and without interruptions; 90.7% considered that the specialist answered their questions clearly; 80.9% responded that the specialist resolved their doubts about health-related self-care; 75% reported that the specialist performed a clinical examination. In addition, 50% did not receive any prescription, 47.2% with prescription received all medicines at the hospital pharmacy; and 2.8% did not receive prescribed medicines, since the pharmacy was out of stock. Most patients reported that the cleanliness of the hospital was good (78.3%); 83.0% of users attending outpatient consultations at IMSS hospitals were satisfied with the service received.

Table 3 depicts the socio-demographic, clinical characteristics and healthcare experiences of users who had inpatient surgery. Out of the 528 users, most were women (60.3%); 29.3% of the respondents were younger than 36 years of age, 53.2% were between 36 and 64 years and 17.5% were older; with completed secondary school or higher education (71.9%); and were residents of the region of stratum four and six, corresponding to medium and high socio-economic levels. Additionally, 82.6% underwent surgery at secondary level hospitals, and the most frequent surgery indications were diseases of the digestive (24.6%) and genitourinary (13.7%) systems.

**Table 3:** Socio-demographic, clinical characteristics and healthcare experiences of users with surgical care

Variables	Total *n* = 528
	Weighted %
Socio-demographic and clinical characteristics	
Women	60.3
Age groups	
≤ 35 years	29.3
> 35 & ≤ 44 years	20.6
> 44 & ≤64 years	32.6
≥ 65 years	17.5
Schooling	
Incomplete elementary school or without formal education	11.5
Compete elementary school	16.6
Complete secondary school or higher	71.9
Region of residence according to the socio-economic level	
Stratum 1 (lowest socio-economic level)	1.6
Stratum 2	12.6
Stratum 3	5.1
Stratum 4	24.1
Stratum 5	15.6
Stratum 6	20.7
Stratum 7 (highest socio-economic level)	20.3
Level of healthcare in which surgery was performed	
Secondary	82.6
Tertiary	17.4
Cause of surgery	
Diseases of the digestive system	24.6
Diseases of the genitourinary system	13.7
Diseases of the musculoskeletal system and connective tissue	12.8
Injury, poisoning and other consequences of external causes	8.9
Diseases of the eye and adnexa	8.2
Other types of diseases and cesarean section	17.4
Symptoms, signs and abnormal clinical and laboratory findings, not elsewhere classified	14.4
Users experience with surgery	
I. Client focus	
Wait time between recommendation for surgery and actual surgery ≤20 days	71.1
History of surgery postponement(s)	11.1
II. Respect	
Surgeon greeted patient before a surgery	82.2
Surgeon explained the risks and benefits of a surgery	87.2
Surgeon gave clear information to the patient’s relatives	85.8
III. Quality of basic amenities	
Hospital’s cleanliness very good or good	76.4
IV. Quality impact on health	
Patient had complications that required another surgery	13.0
Overall satisfaction	
Satisfied with surgery	86.6

Overall, 71.1% of users waited for ≤20 days between being referred to surgery and undergoing the actual surgery; 11.1% experienced one or more surgery postponements; 82.2% informed that the surgeon greeted them before the operation; 87.2% reported that the surgeon explained the risk and benefits of the surgery; 85.8% responded that the surgeon gave clear information to their relatives; 13% of the patients had surgical complications that required another surgery; 76.4% reported that the cleanliness of the hospital was good; 86.6% of users who underwent inpatient surgery at IMSS hospitals were satisfied with the service received.

Table 4 presents the results of the bivariate analysis and multiple double-weighted Poisson regression analysis to identify user experience related to satisfaction with outpatient consultation. The results of the bivariate analysis show that patients with shorter waiting time, those who were greeted by the specialist, who had an opportunity to talk about health-related concerns, who were listened to without interruption, who received clear answers to their questions, who resolved doubts about their self-care, who had a clinical examination, and who were attended to in a clean hospital were more likely to be satisfied with outpatient consultation in comparison with those who did not (*p* < 0.05). Also, older patients living in the areas of the lowest or highest socio-economic level (region of stratum 1 and 7) and those who received care at the tertiary level hospital more often reported being satisfied with outpatient consultation (p < 0.05).

**Table 4:** User experience related to satisfaction with outpatient consultation (*n* = 6713)

TYPE OF ANALYSIS	Bivariable analysis		Simple double-weighted Poisson regression analysis	Multiple double-weighted Poisson regression analysis
VARIABLES	Satisfied *n* = 5738	Dissatisfied *n* = 975	Crude PR (95% CI)	Adjusted PR (95% CI)
	Weighted %	Weighted %		
Users experience with outpatient consultation				
I. Client focus				
Wait time*				
≤ 30 min	87.8	12.2	**1.15 (1.11; 1.90)**	**1.11 (1.07; 1.14)**
> 30 min	76.1	23.9	Ref.	Ref.
II. Respect				
Specialist greeted patient at the beginning of the consultation*				
Yes	85.8	14.2	**1.21 (1.15; 1.28)**	1.04 (0.99; 1.09)
No	70.7	29.3	Ref	Ref.
Specialist gave the patient an opportunity to talk about health-related concerns*				
Yes	85.1	14.9	**1.25 (1.17; 1.34)**	**1.11 (1.03; 1.19)**
No	67.8	32.2	Ref.	Ref.
Specialist listened to the patient with attention and without interruptions*				
Yes	85.8	14.3	**1.44 (1.32; 1.56)**	**1.16 (1.05; 1.29)**
No	59.7	40.3	Ref.	Ref.
Specialist answered clearly patients’ questions*				
Yes	85.5	14.5	**1.46 (1.34; 1.59)**	**1.17 (1.04; 1.31)**
No	58.6	41.4	Ref.	Ref.
Specialist resolved patients´ doubts about the health-related self-care*				
Yes	86.5	13.5	**1.27 (1.20; 1.34)**	**1.16 (1.11; 1.22)**
No	68.3	31.7	Ref.	Ref.
III. Competent care				
Specialist performed clinical examination*				
Yes	58.5	14.5	**1.13 (1.09; 1.18)**	**1.06 (1.02; 1.11)**
No	75.6	24.4	Ref.	Ref.
IV. Quality of basic amenities				
Hospital’s cleanliness*				
Good, very good	87.1	12.9	**1.28 (1.21; 1.34)**	**1.19 (1.14; 1.26)**
Regular, bad or terrible	68.2	31.8	Ref.	Ref.
V. Quality impact on financial risk protection				
Patients received all prescribed medicines in the hospital pharmacy*				
Yes	83.5	16.4	**1.16 (1.03; 1.31)**	1.09 (0.97; 1.21)
No	71.8	28.2	Ref.	Ref.
Did not have any prescription to fulfill	83.2	16.8	**1.16 (1.03; 1.30)**	1.11 (0.99; 1.23)
Covariates				
Patients’ socio-demographic and clinical characteristics				
Men	83.6	16.4	0.99 (0.96; 1.02)	1.00 (0.97; 1.03)
Women	82.8	17.2	Ref.	Ref.
Age groups*				
≤ 35 years	82.9	17.1	Ref.	Ref.
> 35 & ≤ 44 years	81.3	18.7	0.98 (0.93, 1.03)	1.00 (0.97; 1.04)
> 44 & ≤64 years	82,1	17.9	0.99 (0.95, 1.03)	1.01 (0.97; 1.05)
≥ 65 years	86.2	13.8	1.04 (0.99, 1.08)	1.04 (0.99; 1.09)
Schooling				
Incomplete elementary school or without formal education	85.3	14.7	1.04 (0.99; 1.08)	1.02 (0.98; 1.06)
Compete elementary school	84.1	15.9	1.02 (0.99; 1.06)	1.01 (0.97; 1.05)
Complete secondary school or higher	82.2	17.8	Ref.	Ref.
Region of residence*				
Stratum 1	87.2	12.8	**1.07 (1.01; 1.13)**	**1.09 (1.01; 1.17)**
Stratum 2	83.3	16.7	1.02 (0.97; 1.07)	1.00 (0.95; 1.05)
Stratum 3	82,6	17.4	1.01 (0.95; 1.07)	1.03 (0.98; 1.08)
Stratum 4	83.8	16.2	1.02 (0.97; 1.08)	**1.06 (1.01; 1.12)**
Stratum 5	79.1	20.9	0.97 (0.91; 1.03)	1.03 (0.96; 1.10)
Stratum 6	81.8	18.2	Ref.	Ref.
Stratum 7	85.9	14.1	1.05 (0.99; 1.10)	1.04 (0.99; 1.08)
Level of healthcare*				
Secondary	81.8	18.2	Ref.	Ref.
Tertiary	89.4	10.6	**1.09 (1.07; 1.12)**	**1.08 (1.05; 1.11)**
Type of specialty consultation				
Medical-surgical consultation	82.1	17.9	1.03 (0.99; 1.05)	1.02 (0.99; 1.06)
Clinical specialties consultation	84.2	15.8	Ref.	Ref.
Cause of specialty consultation				
Neoplasms	83.8	16.2	1.04 (0.97; 1.13)	1.05 (0.98; 1.12)
Endocrine, nutritional and metabolic diseases	87.3	12.7	**1.09 (1.01; 1.17)**	1.07 (0.99; 1.14)
Mental, behavioural disorders and diseases of the nervous system	86.3	13.7	1.08 (0.99; 1.17)	1.08 (0.98; 1.19)
Diseases of the eye and adnexa	81.2	18.8	1.01 (0.93; 1.10)	1.00 (0.93; 1.08)
Diseases of the respiratory system and diseases of the ear	83.9	16.1	1.05 (0.98; 1.14)	1.04 (0.96; 1.13)
Diseases of the circulatory system	83.6	16.4	1.04 (0.96; 1.13)	1.01 (0.93; 1.09)
Diseases of the digestive system	81.1	18.9	1.01 (0.92; 1.11)	1.00 (0.91; 1.10)
Diseases of the musculoskeletal system and connective tissue	78.9	21.1	0.98 (0.91; 1.07)	1.01 (0.93; 1.10)
Diseases of the genitourinary system	84.6	15.4	1.05 (0.98; 1.13)	**1.07 (1.004; 1.14)**
Pregnancy, childbirth and the puerperium	85.2	14.8	1.06 (0.98; 1.15)	1.04 (0.97; 1.11)
Injury, poisoning and other consequences of external causes	80.1	19.9	0.99 (0.93; 1.07)	1.02 (0.96; 1.09)
Other diseases	85.5	14.5	1.06 (0.98; 1.16)	1.07 (0.98; 1.15)
Factors influencing health status and contact with health services	83.6	16.4	1.04 (0.96; 1.12)	1.02 (0.95; 1.10)
Symptoms, signs and abnormal clinical and laboratory findings, not elsewhere classified	80.2	19.8	Ref.	Ref.

*p < 0.05 in bivariate analysis. PR: prevalence ratios; CI: confidence interval. The bold values highlight the statistically significant adjusted PR

To build the multiple Poisson regression model we used all conceptually relevant variables. The coefficients represent prevalence ratios (PR); their interpretation is the same as for risk ratios. The analysis revealed that the following patient experiences increase the probability of satisfaction: shorter waiting time (adjusted PR:1.11; 95%CI:1.07–1.14), an opportunity to talk about health-related concerns (adjusted PR:1.11; 95%CI:1.03–1.19), being listened to without interruption (adjusted PR:1.16; 95%CI:1.05–1.29), receiving clear answers to their questions (adjusted PR:1.17; 95%CI:1.04–1.31), perceiving that their doubts about self-care were resolved (adjusted PR:1.16; 95%CI:1.11–1.22), being examined (adjusted PR:1.06; 95%CI:1.02–1.11) and experiencing hospital cleanliness (adjusted PR:1.19; 95%CI:1.14–1.26). Additionally, several patient socio-demographic and clinical characteristics increased the probability of patient satisfaction, including being a resident of stratum 1 (lowest socioeconomic level) and 4 (medium socioeconomic level) (adjusted PR:1.09; 95%CI:1.01–1.17 and PR:1.06; 95%CI:1.01–1.12, respectively) and having a consultation due to a disease of the genitourinary system (adjusted PR:1.07; 95%CI:1.01–1.14).

Table 5 presents the results of the bivariate analysis and multiple double-weighted Poisson regression analysis to identify user experiences related to satisfaction with surgery. In the bivariate analysis, patients with incomplete elementary school or without formal education, those without prior postponement(s) of the surgery, those who were greeted by the surgeon before the surgery, those whose relatives received clear information about the surgery, and those who did not have complications and received care at a clean hospital were more likely to report being satisfied with the surgery in comparison with those who did not (*p* < 0.05).

**Table 5:** User experience related to satisfaction with surgical care (n = 528)

TYPE OF ANALYSIS	Bivariable analysis		Simple double-weighted Poisson regression analysis	Multiple double-weighted Poisson regression analysis
VARIABLES	Satisfied *n* = 457	Dissatisfied *n* = 71	Crude PR (95% CI)	Adjusted PR (95% CI)
	Weighted %	Weighted %		
Users experience with a surgery				
I. Client focus				
Waiting time				
≤ 20 days	86.6	13.4	1.05 (0.93; 1.18)	1.04 (0.93; 1.17)
≥ 21 days	84.1	15.9		Ref.
Previous surgery postponement(s)*				
Yes	67.7	32.3	Ref.	Ref.
No	88.1	11.9	1.26 (0.99; 1.60)	**1.24 (1.002; 1.54)**
II. Respect				
Surgeon greeted patient before a surgery*				
Yes	88.6	11.4	**0.82 (0.69; 0.98)**	1.10 (0.94; 1.30)
No	73.1	26.9	Ref.	Ref.
Surgeon explained the risks and benefits of a surgery				
Yes	87.2	12.8	1.10 (0.95; 1.27)	0.99 (0.85; 1.15)
No	76.5	23.5	Ref.	Ref.
Surgeon gave clear information to the patient’s relatives*				
Yes	89.9	10.1	**1.39 (1.12; 1.72)**	1.21 (0.99; 1.49)
No	65.8	34.2	Ref.	Ref.
III. Quality of basic amenities				
Hospital’s cleanliness*				
Very good or good,	89.4	10.6	**1.21 (1.03; 1.41)**	1.13 (0.98; 1.30)
Regular, bad or terrible	74.3	25.7	Ref.	Ref.
IV. Quality impact on health				
Patient had complications that required another surgery*				
Yes	63.5	36.5	Ref.	Ref.
No	89.2	10.8	**1.38 (1.08; 1.76)**	**1.30 (1.03; 1.64)**
Covariates				
Patients’ socio-demographic and clinical characteristics				
Men	85.8	14.2	1.00 (0.91; 1.11)	1.00 (0.93; 1.09)
Women	85.9	14.1	Ref.	Ref.
Age groups				
≤ 35 years	84.3	15.7	Ref.	Ref.
> 35 & ≤ 44 years	81.0	19.0	0.94 (0.78; 1.13)	0.97 (0.84; 1.13)
> 44 & ≤64 years	86.8	13.2	1.05 (0.94; 1.18)	1.04 (0.94; 1.14)
≥ 65 years	92.3	7.7	1.11 (0.98; 1.25)	1.08 (0.93; 1.25)
Schooling*				
Incomplete elementary school or without formal education	93.9	6.1	**1.14 (1.05; 1.24)**	1.07 (0.98; 1.17)
Compete elementary school	89.8	10.2	1.09 (0.99; 1.21)	1.07 (0.98; 1.17)
Complete secondary school or higher	82.0	18.0	Ref.	Ref.
Region of residence				
Stratum 1	93.1	6.9	1.11 (0.90; 1.37)	**1.23 (1.01; 1.51)**
Stratum 2	77.9	22.1	Ref.	Ref.
Stratum 3	92.9	7.1	**1.18 (1.03; 1.35)**	1.13 (0.97; 1.32)
Stratum 4	91.4	8.6	**1.16 (1.02; 1.32)**	**1.19 (1.03; 1.38)**
Stratum 5	83.1	16.9	0.99 (0.81, 1.22)	1.10 (0.90; 1.35)
Stratum 6	82.1	17.9	1.04 (0.87; 1.24)	1.06 (0.90; 1.26)
Stratum 7	87.8	12.2	1.08 (0.91, 1.27)	1.12 (0.96; 1.30)
Level of healthcare				
Secondary	85.1	14.9	Ref.	Ref.
Tertiary	89.7	10.3	1.01 (0.90; 1.14)	1.04 (0.94; 1.15)
Cause of surgery				
Diseases of the digestive system	87.8	12.2	1.15 (0.92; 1.44)	**1.19 (1.005; 1.41)**
Diseases of the genitourinary system	78.2	21.8	Ref.	Ref.
Diseases of the musculoskeletal system and connective tissue	87.4	12.6	1.18 (0.94; 1.48)	1.15 (0.95; 1.38)
Diseases of the eye and adnexa	90.6	9.4	**1.24 (1.01; 1.52)**	1.17 (0.95; 1.45)
Injury, poisoning and other consequences of external causes	75.4	24.6	1.03 (0.76; 1.39)	1.03 (0.85; 1.24)
Other types of diseases and cesarean section	87,5	12.5	1.20 (0.97; 1.47)	1.13 (0.94; 1.34)
Symptoms, signs and abnormal clinical and laboratory findings, not elsewhere classified	90.2	9.8	1.20 (0.97; 1.48)	1.13 (0.95; 1.35)

*p < 0.05 in bivariate analysis. PR: prevalence ratios; CI: confidence interval. The bold values highlight the statistically significant adjusted PR

The multiple Poisson regression analysis revealed that having the surgery without prior postponement(s) (adjusted PR:1.24; 95%CI:1.002–1.54) and without complications (adjusted PR:1.30; 95%CI:1.03–1.64) increased the probability of patient satisfaction with surgery. Also, being a resident of stratum 1 (lowest level of welfare) and 4 (medium level of welfare) (adjusted PR:1.23; 95%CI:1.01–1.51 and PR:1.19; 95%CI:1.03–1.38, respectively) and having surgery due to a digestive system disease (adjusted PR:1.19; 95%CI:1.005–1.41) were additional factors that increased the probability of patient satisfaction with surgery.

The results of the sensitivity analyses using multilevel regressions with ENSAT 2017 and 2016 data revealed similar associations between the same independent and dependent variables in terms of direction and statistical significance (Additional file 1). In addition, in 2016, satisfaction with surgery increased when the surgeons explained the risks and benefits of surgery to the patients and gave clear information to their relatives.

### Discussion

The present study found that lengthy waiting time and lack of hospital cleanliness constituted common patient negative experiences with specialty consultations and surgical care. An additional concern was the lack of clinical examination during the consultations. Notably, shorter waiting times, health provider courtesy, good communication, clinical examination, and hospital cleanliness were associated with patient satisfaction with specialty consultations; while having the surgery without prior postponement(s) and without complications increased the probability of patient satisfaction with surgical care. To the best of our knowledge, this finding is novel for Mexico and Latin America, as the association of patient experiences with hospital care and their satisfaction was not previously investigated in these countries.

Positive patient experiences with healthcare and satisfaction are important indicators of healthcare quality. The present study found that user experience is a major predictor of satisfaction in the multiple regression model that included patient socio-demographic and clinical factors. Evaluating user experience is important because positive experiences are associated with better health outcomes, and negative experiences can guide healthcare improvement initiatives [28]. A systematic review of 55 studies found consistent positive associations between patient experience and adherence to recommended clinical practice, medication and preventive activities (e.g., use of screening services and immunization), among others [28].

The high levels of satisfaction with outpatient consultations and inpatient surgical care (> 80%) observed in our study has been a frequent finding in Mexico and other low and middle-income countries [20–22, 24]. Such a finding should be interpreted with caution because rather than signaling high quality of care, this result can be influenced by low expectations and low health literacy of people unaware of the quality of healthcare that the health system should provide them with. Also, the “social desirability” factor might influence positive opinions. Therefore, to obtain more constructive insights about patients’ expectations and satisfaction, health providers should build up the health literacy of the population they serve [29].

In both consultation with specialist and surgery, the higher percentage of positive patient experiences were observed in the “respect” domain of the framework of the Lancet Global Health Commission on High-Quality Health Systems. This domain included health provider courtesy and clear communication and was reflected in the fact that over 80% of patients reported that the specialists greeted them, let them talk about their health-related concerns, listened without interruption, gave clear answers to their questions and resolved their doubts. Also, the experiences related to clear communication during the consultation with the specialist were associated with an increased probability of patient satisfaction. These results are consistent with studies from other countries [13, 30–32]. In addition, satisfaction with effective patient-provider communication is associated with better patient adherence to the providers’ recommendations, and better functional and psychological health [33, 34]. Therefore, improving patient-provider communication is crucial and can be achieved through training health professionals in communication skills.

User experience in the “client focus domain” that included waiting time for outpatient consultation in hospital outpatient area and time elapsed between referral to the surgery and surgery realization, should improve. We observed that 40.8% of patients reported that waiting for specialist consultation was longer than 30 min and one out of three patients had to wait for more than 20 days to undergo surgery. Interviews with patients in 41 countries identified prompt attention as the most critical characteristic of the non-clinical quality of care [15]. The problems of lengthy waiting time for consultation or elective surgery is an essential concern in both high and low-income countries [35]. The imbalance in demand and supply is the usual explanation for long waiting times. The imbalance occurs if patient demand exceeds the supply of health care providers and hospital beds [36]. The probability of patient satisfaction is higher if they wait less than 30 min [37]. To address long waiting times, most member states of the Organisation for Economic Cooperation and Development monitor national waiting times and have a national waiting time care guarantee; yet to improve waiting time, healthcare systems should invest in the strengthening of their supply capacity [36].

One out of ten patients that underwent surgery reported that the surgery had been postponed before. At the same time, not having the surgery postponed was one of the factors associated with patient satisfaction. These results are consistent with those from the analysis of complaints after postponed surgeries submitted to the National Commission of Medical Arbitration in Mexico [38] and the results from other countries that revealed the negative impact of postponements on the emotional state of patients and their families [39, 40].

A quarter of the patients who attended the outpatient consultation did not have a clinical examination that forms the essential part of “competent care”. This finding is consistent with those from other low and-middle income countries, as revealed by the Lancet Global Health Commission on High-Quality Health Systems. In our study, clinical examination increased the probability of patient satisfaction. Therefore, healthcare providers training should focus on “competent care” including clinical examination in each consultation.

Two out of every ten patients considered that the IMSS hospitals lack good cleanliness. According to the World Health Organization, hospital cleanliness is an essential feature of Health System Responsiveness. However, similar to our findings, several studies from other low and middle-income countries found that their health facilities lack cleanliness [35, 41]. Also, good hospital cleanliness was associated with patient satisfaction.

Surgery without complications was another factor associated with patient satisfaction. Current evidence on the association of in-hospital health outcomes with patient satisfaction varied among countries and studies. In Germany [42], a survey of 39 hospitals identified the outcome of treatment as the main determinate of patient satisfaction. A study from 15 hospitals in the State of Hidalgo, Mexico [22], reported that patient dissatisfaction was associated with the presence of complications. Also, a study of medical complaints due to surgical complications in Mexico revealed that the absence of actions or inappropriate actions at the facility level (e.g., ignoring complaints or lack of information by health provider), were associated with a higher probability of complaints to the National Commission of Medical Arbitration [43]. Several other studies from the United States found that postoperative complications had no impact on overall patient satisfaction ranking [44, 45].

The ENSAT uses a single question to evaluate patient satisfaction. The current evidence supports that a single question might be a useful survey tool for patient satisfaction measurement [46]. Concurrently, the complexity of the consumer satisfaction construct and the limitations of a single overall question (e.g., acquiescent response, social desirability bias, as well as the need to reduce the entire history of interaction with a service to a single rating) are widely recognized [47–51]. Consequently, multiple researchers suggest using jointly a single question and specific patient experiences measures to provide insights on patient satisfaction and quality of care improvement [47–51]. Unlike to a single question of overall satisfaction that requires the consumer to consider all aspects of the service to come to a final decision, the measurement of experiences of care are more precise and reflect judgments about specific events or situations.

The results of the present study contribute to the knowledge of how the specific experiences with hospital healthcare are related to the overall satisfaction in the context of Mexican culture and support the simultaneous use of a single question and patient experiences measures.

The study has several strengths including: (1) focus on user experience that is aligned with the global trend of promoting patient-centered care; specifically, the study adds relevant information to the still limited evidence on specific patients experiences associated with satisfaction with hospital outpatient and surgical care in a middle income country. (2) Use of the 2018 Lancet Global Health Commission on High-Quality Health Systems conceptual framework to guide the study analysis. (3) The representativeness of the population-based survey at national, states and hospital levels. (4) IP weighting that can be considered a strength of this study, as IP weights provide control against possible selection bias arising from missing data. (5) Data from a single health system that comprises the information on which hospital each person went to; this is particularly useful for potential improvement efforts because relevant results can be shared with specific facilities.

At the same time our study has several limitations. First, this study was the analysis of a cross-sectional survey that does not allow for making causal inferences or identifying the direction of the association between the study variables. Second, due to the nature of the study (secondary data analysis), it was limited to the survey information; for instance, ENSAT lacks information on other important patient experiences, such as confidence and trust in healthcare provider, time spent with provider, dignity, and privacy, among others. In addition, the survey does not provide information on patient healthcare expectations and preferences that are important to understand patient satisfaction. These constructs should be included in future ENSAT surveys. Third, due to the characteristics of the sampling frame, there is a high probability of capturing surgery patients with shorter hospital stays since the sampling frame included patients who were discharged immediately after surgery. Finally, the results of our study can be generalizable only to the IMSS hospitals users, as the survey did not include users of the Ministry of Health or private hospitals.

In conclusion, patient satisfaction with outpatient consultations and surgical care may be increased through focusing on improvement strategies aimed at enhancing positive experiences associated with good satisfaction. These include shorter waiting times, health provider courtesy, clear communication and competent care (clinical examination), as well as prevention of surgical complications and improvement of hospital cleanliness. The findings of this study support existing research on the importance of clinical care and positive patient experience for satisfaction; they are the first to show this association in Mexico for hospital care.

## Supplementary information

**Additional file 1: Table S1.** User experience related to satisfaction with outpatient consultation (*n* = 6713 patients nested within 136 hospitals; year 2017). **Table S2.** User experience related to satisfaction with outpatient consultation (*n* = 10,328 patients nested within 130 hospitals; year 2016). **Table S3.** User experience related to satisfaction with surgical care (*n* = 1082 patients nested within 124 hospitals; year 2016).

## Data Availability

The data supporting the results reported in this article is available at the IMSS webpage at: http://www.imss.gob.mx/encuesta-nacional/sistema-integral-de-medicion-de-la-satisfaccion-de-usuarios

## Abbreviations

CI: confidence intervals
ENSAT: National Satisfaction Survey
ICD-10: International Classification of Diseases 10th revision
IMSS: Mexican Institute of Social Security
PR: prevalence ratios
