# Supplementary material for: User experience and satisfaction with specialty consultations and surgical care in secondary and tertiary level hospitals in Mexico
Source: BMC Health Serv Res. 2019 Nov 21;19:872. doi: 10.1186/s12913-019-4706-9 (PMC6873740; doi:10.1186/s12913-019-4706-9)
Supplement: Supplementary file 1 — Additional file 1: Table S1. User experience related to satisfaction with outpatient consultation (n = 6713 patients nested within 136 hospitals; year 2017). Table S2. User experience related to satisfaction with outpatient consultation (n = 10,328 patients nested within 130 hospitals; year 2016). Table S3. User experience related to satisfaction with surgical care (n = 1082 patients nested within 124 hospitals; year 2016). [file 12913_2019_4706_MOESM1_ESM.docx]

**Additional file 1**

**Table S1. User experience related to satisfaction with outpatient consultation** (n=6713 patients nested within 136 hospitals; year 2017)

| **TYPE OF ANALYSIS**    **VARIABLES** | **Multilevel double-weighted logistic regression analysis** |
| --- | --- |
|  | **Adjusted Odds Ratios**  **(95% CI)** |
|  |  |
| **Users experience with outpatient consultation** |  |
| **I. Client focus** |  |
| Wait time* |  |
| ≤30 minutes | **1.97 (1.60; 2.42)** |
| >30 minutes | Ref. |
| **II. Respect** |  |
| Specialist greeted patient at the beginning of the consultation*  Yes  No | 1.22 (0.93; 1.59)  Ref. |
| Specialist gave the patient an opportunity to talk about health-related concerns*  Yes  No | **1.56 (1.14; 2.14)**  Ref. |
| Specialist listened to the patient with attention and without interruptions*  Yes  No | **1.87 (1.26; 2.76)**  Ref. |
| Specialist answered clearly patients’ questions*  Yes  No | **1.73 (1.13; 2.66)**  Ref. |
| Specialist resolved patients´ doubts about the health-related self-care*  Yes  No | **2.13 (1.71; 2.65)**  Ref. |
| **III. Competent care** |  |
| Specialist performed clinical examination*  Yes  No | **1.49 (1.15; 1.93)**  Ref. |
| **IV. Quality of basic amenities** |  |
| Hospital's cleanliness*  Good, very good  Regular, bad or terrible | **2.27 (1.84; 2.81)**  Ref. |
| **V. Quality impact on financial risk protection** |  |
| Patients received all prescribed medicines in the hospital pharmacy*  Yes  No  Did not have any prescription to fulfill | 1.55 (0.91; 2.65)  Ref.  **1.65 (1.01; 2.70)** |
| **Covariates** |  |
| **Patients’ socio-demographic and clinical characteristics** |  |
| Men | 1.02 (0.82; 1.28) |
| Women | Ref. |
| Age groups* |  |
| ≤35 years | Ref. |
| >35 & ≤ 44 years | 1.00 (0.79; 1.27) |
| >44 & ≤64 years | 1.08 (0.82; 1.42) |
| ≥65 years | 1.29 (0.90; 1.85) |
| Schooling |  |
| Incomplete elementary school or without formal education | 1.19 (0.83; 1.70) |
| Compete elementary school | 1.07 (0.81; 1.42) |
| Complete secondary school or higher | Ref. |
| Region of residence* |  |
| Stratum 1 | 1.75 (0.76; 4.06) |
| Stratum 2 | 0.99 (0.63; 1.55) |
| Stratum 3 | 1.10 (0.79; 1.52) |
| Stratum 4 | **1.42 (1.03; 1.94)** |
| Stratum 5 | 1.24 (0.81; 1.90) |
| Stratum 6 | Ref. |
| Stratum 7 | 1.29 (0.90; 1.86) |
| Level of healthcare* |  |
| Secondary | Ref. |
| Tertiary | **1.99 (1.54; 2.56)** |
| Type of specialty consultation |  |
| Medical-surgical consultation | 1.26 (0.97; 1.63) |
| Clinical specialties consultation | Ref. |
| Cause of specialty consultation |  |
| Neoplasms | 1.39 (0.91; 2.14) |
| Endocrine, nutritional and metabolic diseases | 1.67 (0.97; 2.87) |
| Mental, behavioural disorders and diseases of the nervous system | 1.73 (0.80; 3.77) |
| Diseases of the eye and adnexa | 1.09 (0.69; 1.72) |
| Diseases of the respiratory system and diseases of the ear | 1.39 (0.79; 2.44) |
| Diseases of the circulatory system | 1.17 (0.66; 2.06) |
| Diseases of the digestive system | 1.03 (0.59; 1.81) |
| Diseases of the musculoskeletal system and connective tissue | 1.14 (0.69; 1.88) |
| Diseases of the genitourinary system | **1.56 (1.06; 2.30)** |
| Pregnancy, childbirth and the puerperium | 1.42 (0.88; 2.29) |
| Injury, poisoning and other consequences of external causes | 1.22 (0.78; 1.90) |
| Other diseases | 1.54 (0.87; 2.74) |
| Factors influencing health status and contact with health services | 1.16 (0.72; 1.89) |
| Symptoms, signs and abnormal clinical and laboratory findings, not elsewhere classified | Ref. |

OR: odds ratios; CI: confidence interval. The bold values highlight the statistically significant adjusted OR.

Note 1: Stata did not complete multilevel double-weighted Poisson regression analysis for user experience related to satisfaction with outpatient consultation (with ENSAT 2017 data), notifying that the initial values were not feasible for fitting a full model. Yet, we were able to perform multilevel double-weighted logistic regression analysis.

Note 2: Stata did not complete multilevel double-weighted Poisson regression, neither multilevel double-weighted logistic regression for user experience related to satisfaction with surgical care (with ENSAT 2017 data), notifying that discontinuous region was encountered. This is because of the small number of patients who underwent surgery and who answered ENSAT 2017 per hospital, as there were 528 attended in 115 hospitals.

**Table S2. User experience related to satisfaction with outpatient consultation** (n=10328 patients nested within 130 hospitals; year 2016)

| **TYPE OF ANALYSIS**    **VARIABLES** | **Multilevel double-weighted Poisson regression analysis** |
| --- | --- |
|  | **Adjusted PR**  **(95% CI)** |
|  |  |
| **Users experience with outpatient consultation** |  |
| **I. Client focus** |  |
| Wait time* |  |
| ≤30 minutes | **1.13 (1.11; 1.16)** |
| >30 minutes | Ref. |
| **II. Respect** |  |
| Specialist greeted patient at the beginning of the consultation*  Yes  No | **1.06 (1.02; 1.10)**  Ref. |
| Specialist gave the patient an opportunity to talk about health-related concerns*  Yes  No | **1.15 (1.10; 1.21)**  Ref. |
| Specialist listened to the patient with attention and without interruptions*  Yes  No | **1.15 (1.08; 1.22)**  Ref. |
| Specialist answered clearly patients’ questions*  Yes  No | **1.28 (1.19; 1.38)**  Ref. |
| Specialist resolved patients´ doubts about the health-related self-care*  Yes  No | **1.14 (1.10; 1.19)**  Ref. |
| **III. Competent care** |  |
| Specialist performed clinical examination*  Yes  No | **1.04 (1.02; 1.07)**  Ref. |
| **IV. Quality of basic amenities** |  |
| Hospital's cleanliness*  Good, very good  Regular, bad or terrible | This variable was not available in 2016 ENSAT database for second level of care |
| **V. Quality impact on financial risk protection** |  |
| Patients received all prescribed medicines in the hospital pharmacy*  Yes  No  Did not have any prescription to fulfill | **1.11 (1.03; 1.19)**  Ref.  **1.10 (1.02; 1.18)** |
| **Covariates** |  |
| **Patients’ socio-demographic and clinical characteristics** |  |
| Men | 1.00 (0.98; 1.02) |
| Women | Ref. |
| Age groups* |  |
| ≤35 years | Ref. |
| >35 & ≤ 44 years | 0.98 (0.95; 1.01) |
| >44 & ≤64 years | **0.96 (0.93; 0.98)** |
| ≥65 years | 0.97 (0.94; 1.00) |
| Schooling |  |
| Incomplete elementary school or without formal education | **1.04 (1.01; 1.08)** |
| Compete elementary school | **1.04 (1.02; 1.07)** |
| Complete secondary school or higher | Ref. |
| Region of residence* |  |
| Stratum 1 | 1.01 (0.92; 1.12) |
| Stratum 2 | 0.99 (0.93; 1.06) |
| Stratum 3 | 0.98 (0.91; 1.04) |
| Stratum 4 | 0.96 (0.89; 1.04) |
| Stratum 5 | 1.03 (0.95; 1.10) |
| Stratum 6 | Ref. |
| Stratum 7 | 0.99 (0.92; 1.08) |
| Level of healthcare* |  |
| Secondary | Ref. |
| Tertiary | **1.10 (1.04; 1.13)** |
| Type of specialty consultation |  |
| Medical-surgical consultation | 1.03 (1.002; 1.06) |
| Clinical specialties consultation | Ref. |
| Cause of specialty consultation |  |
| Neoplasms | 1.04 (0.98; 1.11) |
| Endocrine, nutritional and metabolic diseases | 1.05 (0.99; 1.11) |
| Mental, behavioural disorders and diseases of the nervous system | 1.03 (0.98; 1.09) |
| Diseases of the eye and adnexa | **1.08 (1.03; 1.14)** |
| Diseases of the respiratory system and diseases of the ear | 1.01 (0.95; 1.08) |
| Diseases of the circulatory system | **1.06 (1.01; 1.12)** |
| Diseases of the digestive system | 0.99 (0.93; 1.07) |
| Diseases of the musculoskeletal system and connective tissue | 1.02 (0.97; 1.08) |
| Diseases of the genitourinary system | 0.99 (0.94; 1.05) |
| Pregnancy, childbirth and the puerperium | 1.01 (0.96; 1.06) |
| Injury, poisoning and other consequences of external causes | 0.98 (0.94; 1.03) |
| Other diseases | 1.02 (0.95; 1.10) |
| Factors influencing health status and contact with health services | 1.05 (0.98; 1.13) |
| Symptoms, signs and abnormal clinical and laboratory findings, not elsewhere classified | Ref. |

PR: prevalence ratios; CI: confidence interval. The bold values highlight the statistically significant adjusted PR.

.

**Table S3. User experience related to satisfaction with surgical care** (n=1082 patients nested within 124 hospitals; year 2016)

| **TYPE OF ANALYSIS**  **VARIABLES** | **Multilevel double-weighted Poisson regression analysis** |
| --- | --- |
|  | **Adjusted PR**  **(95% CI)** |
|  |  |
| **Users experience with a surgery** |  |
| **I. Client focus** |  |
| Waiting time |  |
| ≤20 days | 1.00 (0.93; 1.07) |
| ≥ 21 days | Ref. |
| Previous surgery postponement(s)*  Yes  No | Ref.  **1.14 (1.01; 1.29)** |
| **II. Respect** |  |
| Surgeon greeted patient before a surgery*  Yes  No | 1.03 (0.90; 1.17)  Ref. |
| Surgeon explained the risks and benefits of a surgery  Yes  No | **1.27 (1.10**; 1.46)  Ref. |
| Surgeon gave clear information to the patient's relatives*  Yes  No | **1.33 (1.16; 1.52)**  Ref. |
| **III. Quality of basic amenities** |  |
| Hospital's cleanliness*  Very good or good,  Regular, bad or terrible | This variable was not available in 2016 ENSAT database for second level of care |
| **IV. Quality impact on health** |  |
| Patient had complications that required another surgery*  Yes  No | Ref.  **1.26 (1.12; 1.42)** |
| **Covariates** |  |
| **Patients’ socio-demographic and clinical characteristics** |  |
| Men | 1.01 (0.93; 1.09) |
| Women | Ref. |
| Age groups |  |
| ≤35 years | Ref. |
| >35 & ≤ 44 years | 1.03 (0.94; 1.13) |
| >44 & ≤64 years | 1.03 (0.96; 1.10) |
| ≥65 years | 0.99 (0.87; 1.13) |
| Schooling* |  |
| Incomplete elementary school or without formal education | 1.04 (0.94; 1.16) |
| Compete elementary school | 0.99 (0.91; 1.07) |
| Complete secondary school or higher | Ref. |
| Region of residence |  |
| Stratum 1 | 1.01 (0.90; 1.15) |
| Stratum 2 | Ref. |
| Stratum 3 | 1.03 (0.87; 1.21) |
| Stratum 4 | 1.02 (0.92; 1.13) |
| Stratum 5 | 1.07 (0.96; 1.19) |
| Stratum 6 | 1.002 (0.91; 1.10) |
| Stratum 7 | 1.07 (0.97; 1.19) |
| Level of healthcare |  |
| Secondary | Ref. |
| Tertiary | **1.11 (1.05; 1.19)** |
| Cause of surgery |  |
| Diseases of the digestive system | 1.11 (0.93; 1.31) |
| Diseases of the genitourinary system | Ref. |
| Diseases of the musculoskeletal system and connective tissue | 1.12 (0.93; 1.36) |
| Diseases of the eye and adnexa | 1.18 (0.97; 1.44) |
| Injury, poisoning and other consequences of external causes | 1.04 (0.85; 1.28) |
| Other types of diseases and cesarean section | 1.12 (0.94; 1.34) |
| Symptoms, signs and abnormal clinical and laboratory findings, not elsewhere classified | 1.10 (0.89; 1.36) |

PR: prevalence ratios; CI: confidence interval. The bold values highlight the statistically significant adjusted PR.
